# Atomically Ordered PdCu Electrocatalysts for Selective and Stable Electrochemical Nitrate Reduction

**DOI:** 10.1021/acsenergylett.3c01672

**Published:** 2023-10-19

**Authors:** Jeonghoon Lim, David A. Cullen, Eli Stavitski, Seung Woo Lee, Marta C. Hatzell

**Affiliations:** † George W. Woodruff School of Mechanical Engineering, Georgia Institute of Technology, Atlanta, Georgia 30332, United States; ‡ Center for Nanophase Materials Sciences, Oak Ridge National Laboratory, Oak Ridge, Tennessee 37831, United States; § National Synchrotron Light Source II, Brookhaven National Laboratory, Upton, New York 11973, United States; ∥ School of Chemical and Biomolecular Engineering, Georgia Institute of Technology, Atlanta, Georgia 30332 United States

## Abstract

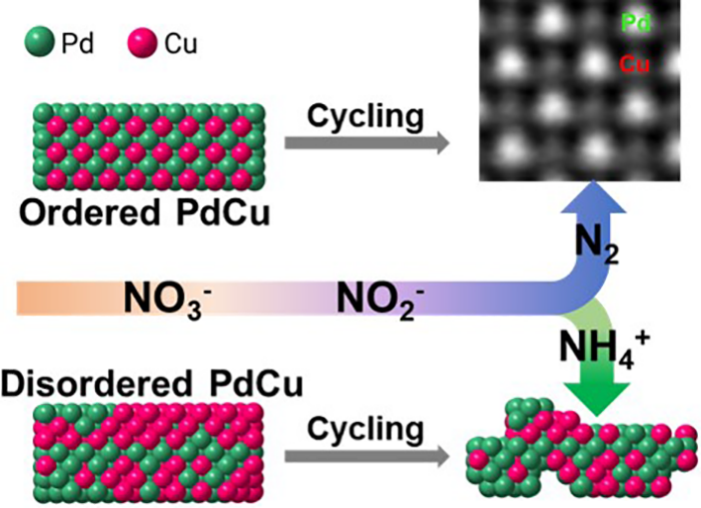

Electrochemical nitrate reduction (NO_3_ RR) has attracted attention as an emerging approach to mitigate nitrate pollution in groundwater. Here, we report that a highly ordered PdCu alloy-based electrocatalyst exhibits selective (91% N_2_), stable (480 h), and near complete (94%) removal of nitrate without loss of catalyst. In situ and ex situ XAS provide evidence that structural ordering between Pd and Cu improves long-term catalyst stability during NO_3_RR. In contrast, we also report that a disordered PdCu alloy-based electrocatalyst exhibits non-selective (44% N_2_ and 49% NH_4_^+^), unstable, and incomplete removal of nitrate. The copper within disordered PdCu alloy is vulnerable to accepting electrons from hydrogenated neighboring Pd atoms. This resulted in copper catalyst losses which were 10× greater than that of the ordered catalyst. The design of stable catalysts is imperative for water treatment because loss of the catalyst adds to the system cost and environmental impacts.

The introduction of anthropogenic nitrogen fertilizers has increased nitrate accumulation in groundwater, negatively affecting human health.^1,2^ The World Health Organization has regulated a maximum contaminant level of 10 mg L^–1^ for NO_3_^–^ and 0.3 mg L^–1^ for NO_2_^–^ in drinking water. Although biological treatment at centralized wastewater treatment facilities is the dominant nitrate treatment approach, there is growing interest in designing technologies to treat nitrate contamination near the source of the pollution. Electrochemical conversion of nitrate pollution is an emerging approach that is designed to meet this need.^3,4^ However, most catalyst examinations related to the electrocatalytic conversion of nitrate focus solely on designing a highly selective catalyst.^1^ While selectivity is important, the use of downstream separations can aid in the tuning of the product purity. Therefore, from a catalysis perspective, material stability may be most important to reduce the cost of the system and prevent additional contamination.^5^

Copper is the state-of-art high activity catalyst for NO_3_RR in neutral and alkaline media.^6^ Whether copper is incorporated in a solid molecular hybrid catalyst or appears as an oxide, the element achieves effective nitrate conversion with good selectivity toward ammonia.^7,8^ However, copper passivation is common in the presence of chloride and copper oxide is destabilized in the presence of ammonium through the formation of a soluble [Cu(NH_3_)_6_]^2+^ complex.^9^ This results in rapid deactivation and loss of Cu, which could inhibit the practical long-term use of Cu for the reduction of nitrate.^6,10^ The most common approach to stabilizing copper is to incorporate copper into an alloy.

Pd–Cu-based alloys have been found to be potentially ideal for NO_3_^–^ conversion to nitrogen gas.^11−15^ The primary advantage of binary PdCu alloys is that Cu strongly absorbs nitrate, reducing NO_3_^–^ to NO_2_^–^ and NO. Then, NO spills from the Cu site to the Pd sites, where NO couples to form N_2_.^16,17^ Here, hydrogenated Pd sites are essential to directly convert NO_2_^–^ to N_2_ and recover metallic Cu from CuO. Cu sites have a tendency to act as an electron donor to reduce NO_3_^–^.^14,18−20^ Despite the clear promise of intermetallic electrocatalyst,^21−25^ few mechanistic studies and long-term stability investigations have been carried out to assess catalyst stability during reduction of nitrate and nitrite.

Here, we examine the electrochemical and catalytic mechanisms that promote the instability of PdCu alloys. We specifically examine how the atomic-level ordering can allow for high degrees of selectivity and stability during NO_3_^–^ conversion. The use of ex situ and in situ XAS provides insight into the mechanism responsible for catalyst loss in disordered PdCu alloys.

To obtain the atomic-level PdCu alloy structure, we first prepared randomly disordered PdCu NPs on a carbon support (denoted D-PdCu/C) and then annealed the catalysts at temperatures ranging from 200 to 600 °C. We characterized the crystal phase by XRD (Supplementary Figure 1). The face-centered cubic (FCC) phase peaks at 41.4°, 48.2°, 70.5°, and 85.3° correspond to (111), (200), (220), and (311) facets. The body-centered cubic (BCC) phase peaks at 29.8°, 42.7°, 52.8°, 62.0°, and 78.0° correspond to (100), (110), (111), (200), and (211) facets. The D-PdCu/C catalyst showed a BCC structure, with the main peak at 42.7° represented for the (110) facet. With increasing annealing temperature, the phase of the FCC(111) and the CsCl structure typed BCC(110) of PdCu emerge. The peaks at higher temperatures were very sharp, mainly because of the larger nanoparticle (NP) size formed by agglomeration. The degree of ordering and crystallinity depend on the annealing temperature, and this was used to control the ratio of FCC and BCC phase structure (F/B). Here, we will focus on examining two highly ordered structures of PdCu obtained by annealing the prepared D-PdCu/C catalyst at 500 °C (denoted O1-PdCu/C) and 600 °C (denoted O2-PdCu/C). O2-PdCu/C showed a higher F/B ratio (0.96) compared to O1-PdCu/C (0.76) and D-PdCu/C (0), as estimated by the two main exposed peaks at 41.5° and 42.8°.

The uniformly distributed D-PdCu NPs on the carbon support showed an average NP size (4.3 nm), as measured by TEM images (Supplementary Figure 2). After annealing at 500 °C, the O1-PdCu/C catalysts showed an average NP size of 5.3 nm. O2-PdCu/C (annealed at 600 °C) exhibited a large average NP size (9.5 nm) and included the largest NPs over 20 nm. As the annealing temperature increased above 500 °C, the catalyst showed severe NP agglomeration within the heating range between 500 and 600 °C, resulting in the transformation of the more FCC structure from the original BCC structure. The reference commercial Pd/C showed 3–5 nm size of nanoparticles (Supplementary Figure 3).

Aberration-corrected bright-field (BF) and high-angle annular dark-field (HAADF) scanning transmission electron microscopy (STEM) images and energy-dispersive X-ray spectroscopy (EDS) maps in Figure 1 clearly demonstrate the ordered phase at the atomic level of O1-PdCu NPs. Representative small NPs and large NP showed both ordered cubic CsCl structure type BCC(110) phases. The alternating contrast between atomic columns in the HAADF-STEM image is consistent with atomic ordering (Figure 1j,k), as confirmed by the EDS mapping results in Figure 1l that display the alternating atomic columns of Pd (green) and Cu (red). The chemical composition of the pristine O2-PdCu/C, O1-PdCu/C and D-PdCu/C catalysts showed a ratio close to 1:1 for Pd and Cu, measured by ICP-MS (Supplementary Table 1).

**Figure 1:**
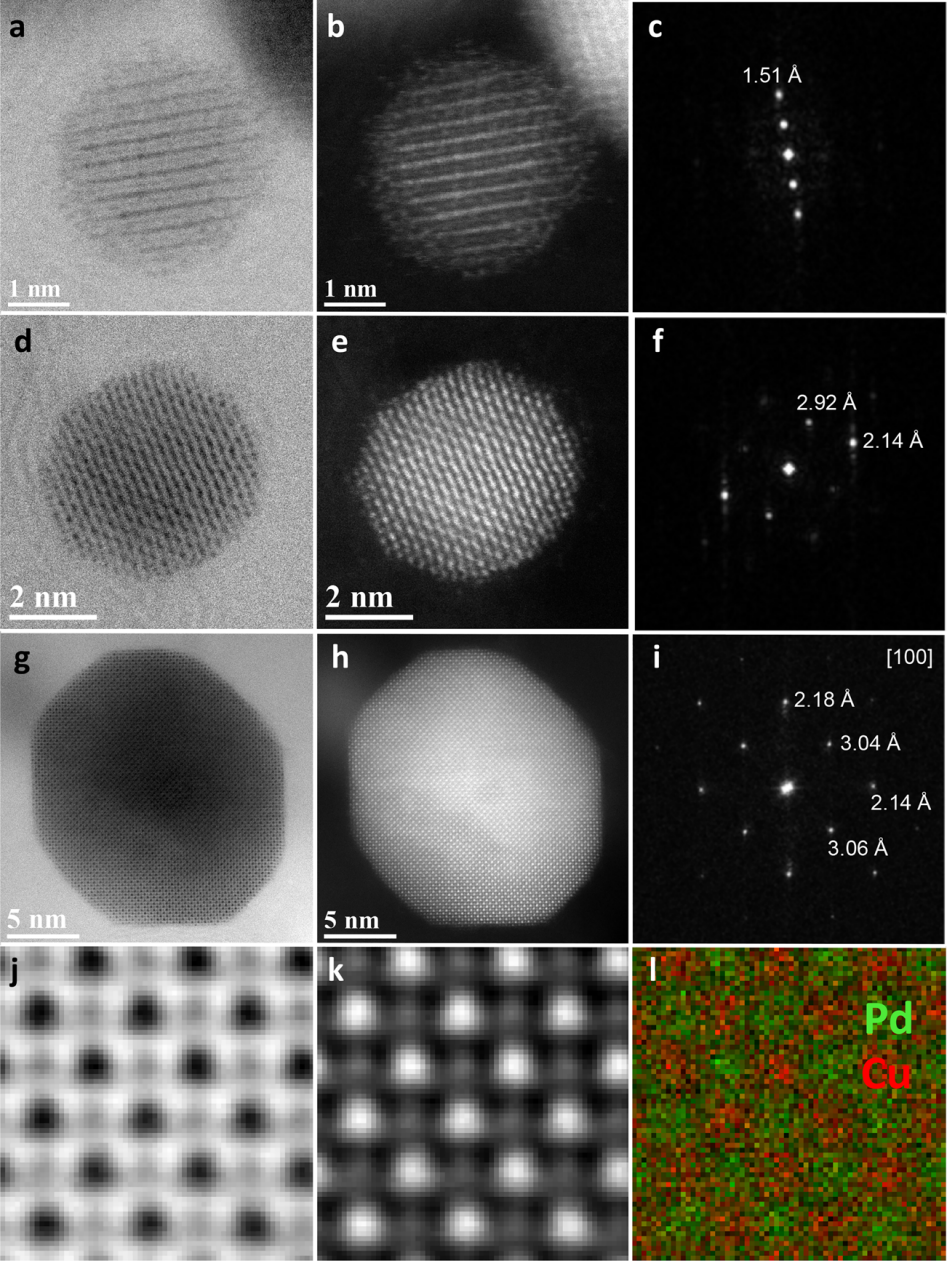
Morphology of O1-PdCu/C (annealed at 500 °C). HRTEM, STEM, fast Fourier transform (FFT) images of O1-PdCu NPs (a–i). Atomically well-ordered structure of Pd and Cu atoms in HRTEM, STEM and EDS mapping results (j–l).

Next, we will evaluate the electrocatalytic performance of nitrate (NO_3_RR) and nitrite reduction (NO_2_RR) (Figure 2a,b), with the activity reported in terms of a partial current density collected at −0.5 V_*RHE*_ (Supplementary Figure 4). Commercial Pd/C showed the highest hydrogen evolution reaction (HER) activity (5.53 mA cm^–2^) as well as NO_3_RR activity (9.03 mA cm^–2^). D-PdCu/C exhibited lower HER activity (3.90 mA cm^–2^) than commercial Pd/C because Cu prohibits the proton adsorption necessary for HER. The D-PdCu/C catalyst showed higher NO_3_RR activity (15.5 mA cm^–2^) than commercial Pd/C. O1-PdCu/C exhibited the highest NO_3_RR activity (20.2 mA cm^–2^) and an obvious reduction peak of NO_3_^–^ around −0.5 V_*RHE*_, while the HER activity (4.13 mA cm^–2^) was slightly higher than that of D-PdCu/C (3.90 mA cm^–2^). This indicates that the atomically ordered structure of Pd and Cu showed a higher NO_3_RR performance due to the creation of multiple active sites on the exposed Pd and Cu atoms, as well as due to electronic modifications. The O2-PdCu/C catalyst showed the lowest current density toward both HER (0.86 mA cm^–2^) and NO_3_RR (1.06 mA cm^–2^); however, this was largely attributed to the size of NP that significantly reduced the ECSA.

**Figure 2:**
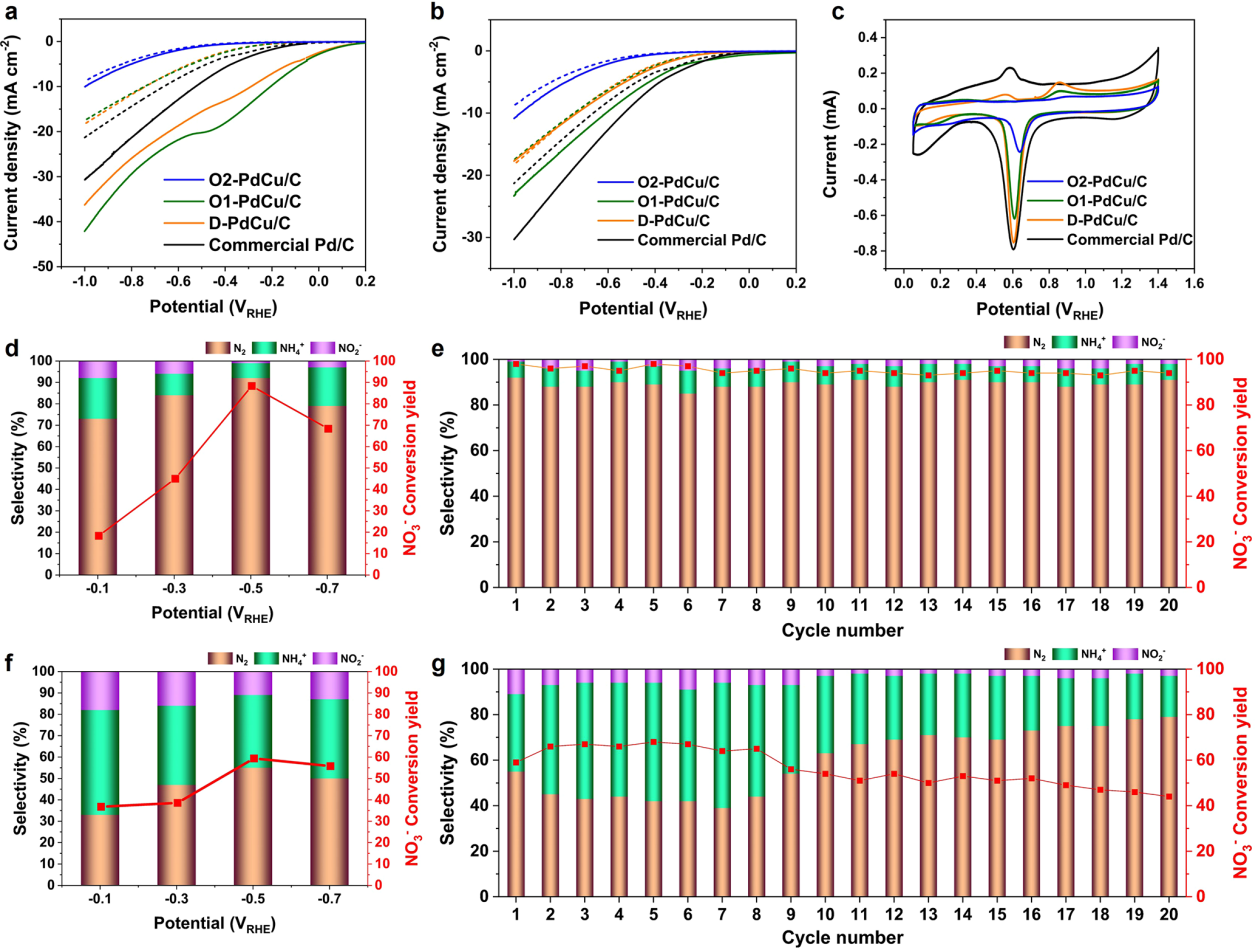
Electrocatalytic performance of (a) NO_3_RR and (b) NO_2_RR. Blank LSV curves (dashed line) for HER were collected in Ar-purged 0.05 M Na_2_SO_4_ electrolyte in the absence of nitrate and nitrite ions. NO_3_RR and NO_2_RR activity (solid line) were measured in Ar-purged 0.05 M Na_2_SO_4_ + 20 mM NO_3_^–^ and 0.05 M Na_2_SO_4_ + 2 mM NO_2_^–^. (c) PdO reduction method to estimate ECSAs of the O2-PdCu/C, O1-PdCu/C, D-PdCu/C, and commercial Pd/C catalysts. O1-PdCu/C: (d) NO_3_^–^ conversion yield and N_2_/NH_4_^+^ selectivity, and (e) stability. D-PdCu/C: (f) NO_3_^–^ conversion yield and N_2_/NH_4_^+^ selectivity, and (g) stability. Each measurement was 24 h of electrolysis in an Ar purged 0.05 M Na_2_SO_4_ + 100 ppm of NO_3_^–^–N electrolyte.

Regarding nitrite reduction, commercial Pd/C unsurprisingly showed the best NO_2_RR performance (8.84 mA cm^–2^) because the Pd catalyst is favorable for NO_2_RR.^26−28^ The D-PdCu/C demonstrated indistinguishable NO_2_RR activity (4.44 mA cm^–2^) and HER activity (3.90 mA cm^–2^). The larger O2-PdCu/C catalyst showed the lowest current density for the NO_2_RR (1.12 mA cm^–2^). Overall the O2-PdCu/C catalyst had nearly identical performance for nitrate and nitrite reduction (Figure 2a,b). We should note that we estimated the ECSAs for all catalysts using PdO reduction area measurement and TEM analysis, and summarize the values in Supplementary Table 2.

The O1-PdCu/C catalyst showed the highest NO_3_^–^ conversion (98%) at −0.5 V_*RHE*_ (Figure 2d), which is in agreement with the LSV curves for the NO_3_RR (Figure 2a). O1-PdCu/C produced the highest N_2_ selectivity (92%) at 0.5 V_*RHE*_ (Figure 2d). Thus, the atomically ordered structure of Pd and Cu is favorable for electrochemical denitrification, and not for NH_4_^+^ synthesis. The D-PdCu/C catalyst exhibited 59.4% of NO_3_^–^ conversion with 55% of the formed products being N_2_ and 34% of the formed products being NH_4_^+^ (Figure 2f). Disordered PdCu NPs have more Cu clusters on the surface than well-ordered PdCu NPs. Cu clusters are known to catalyze and guide a pathway from NO_3_^–^ to NH_4_^+^ rather than N_2_.

During electrolysis, we connected in situ mass spectrometry with the sealed chamber to verify gaseous products (Supplementary Figure 5–8). The O1-PdCu/C and commercial Pd/C electrodes both released H_2_ and N_2_ as the main gaseous products, but no N_2_O gas is detected. It should be noted that the D-PdCu/C electrode exhibited not only H_2_ and N_2_ as the main gaseous products, but also generated N_2_O gas. This indicated that Cu clusters placed far from Pd sites on the surface may be responsible for the production of N_2_O gas. The Cu sites in the highly ordered structure (O1-PdCu/C) have a close connection with their neighboring Pd atoms, which allows for the reduction of adsorbed N_2_O to N_2_, resulting in high N_2_ selectivity.

Long-term electrolysis is necessary to achieve a practical application.^6,16^ O1-PdCu/C electrode showed outstanding stability with a uniform NO_3_^–^ conversion (93–98%) and N_2_ selectivity (85–92%) over 20 cycles of repeated electrolysis tests for 480 h electrolysis. The final cycle electrolysis of O1-PdCu/C showed excellent NO_3_^–^ conversion (94%) and N_2_ selectivity (91%). On the other hand, the D-PdCu/C electrode exhibited a dynamic change in terms of the NO_3_^–^ conversion and N_2_/NH_4_^+^ selectivity. The initial 8 cycles showed a slight enhancement in the conversion of NO_3_^–^ (59% → 65%) and NH_4_^+^ selectivity (34% → 49%). There is also a slight decrease in selectivity for N_2_ (55% → 44%). This finding implies that surface Cu atoms could undergo an oxidation process more easily with the disordered catalyst during the NO_3_RR electrolysis. Prior reports have shown that oxidized copper has excellent NH_4_^+^ selectivity and NO_3_^–^ conversion.^8,29^ From 8 to 20 cycles, we found a continuous decay of NO_3_^–^ conversion (65% → 44%) and NH_4_^+^ selectivity (49% → 18%) while there is an enhancement of N_2_ selectivity (44% → 79%). For the D-PdCu/C catalyst, a dissolution of the oxidized Cu occurred, which leached into the electrolyte. Thus, eventually more Pd surfaces are evolving during the long-term NO_3_RR electrolysis, contributing to the transition in selectivity from NH_4_^+^ to N_2_. We proposed a mechanism for O1-PdCu/C and D-PdCu/C during long-term electrochemical NO_3_ RR in Supplementary Figure 9.

We examine the excellent structure stability of O1-PdCu/C through ICP-MS (Supplementary Table 3) and STEM results (Supplementary Figure 10). After continuous electrolysis for up to 20 cycles, we measured the concentration of Cu and Pd in the electrolytes. D-PdCu/C represented not only a higher Cu leaching concentration (547 ppb), but also a higher Pd leaching concentration (18.2 ppb) compared to O1-PdCu/C (Cu: 42.3 ppb, Pd: 4.64 ppb, Supplementary Table 3). The O1-PdCu NP maintained the ordered structure well after 20 repeated cycles (Supplementary Figure 10). These findings demonstrated that O1-PdCu/C showed highly efficient NO_3_^–^ conversion and excellent N_2_ selectivity with outstanding lifetimes in electrochemical NO_3_RR operations (Supplementary Table 4). We performed X-ray absorption spectroscopy (XAS) to examine changes in the oxidation states and coordination environment near the Pd and Cu atoms.^37^

We performed X-ray absorption spectroscopy (XAS) to examine changes in the oxidation states and coordination environment near the Pd and Cu atoms. By annealing at 500 °C to synthesize O1-PdCu/C from D-PdCu/C, the Cu–Cu coordination number (CN) was decreased 6 to 4.8, and Pd–Cu CN was increased 2.4 to 3.4 (Supplementary Table 5 and Table 6). This indicated that more Cu clusters are placed on the surface of D-PdCu/C compared to O1-PdCu/C and phase segregation with a surface atom rearrangement was occurred for more Pd atoms on the surface of O1-PdCu/C.^30−32^ We first demonstrated the role of PdCu ordered structure during the NO_3_RR through ex situ and in situ XAS measurements by using our customized cell. In situ measurements show the oxidation of Cu clusters in D-PdCu/C compared to Cu sites in O1-PdCu/C (Supplementary Figures 11 and 12). This is ascribed to poor coordination with hydrogenated Pd sites. This is because Cu sites which provide electrons to reduce NO_3_^–^ to NO_2_^–^ become more oxidized, but neighboring hydrogenated Pd atoms are easily accessible and reduced to lower Cu oxidation states.^14,33,34^ Using negative potentials from 0.1 to −0.7 V_*RHE*_, the XANES spectra showed a trend for CuOx reduction to metallic Cu regardless of catalysts or the existence of NO_3_^–^ (Figure 3a,d). This finding concluded that the applied negative reduction potentials are more dominant than the oxidation of the Cu sites, as they provide electrons to reduce NO_3_^–^. For ex situ measurement, Pd K-edge spectra indicated that there were no changes for both D-PdCu/C and O1-PdCu/C catalysts before and after the long-term NO_3_RR operations (Figure 3b,c). However, the ex situ characteristics of the Cu K edge after long-term electrolysis (Figure 3e,f), D-PdCu/C showed a significant change due to the higher Cu oxidation states than that of pristine catalyst. However, the O1-PdCu/C spetra did not alter, which implies constant oxidation states before and after long-term electrolysis. These stable states of the O1-PdCu/C demonstrated that intermetallic Pd and Cu nanoparticles have robust stability for long-term NO_3_RR operations.

**Figure 3:**
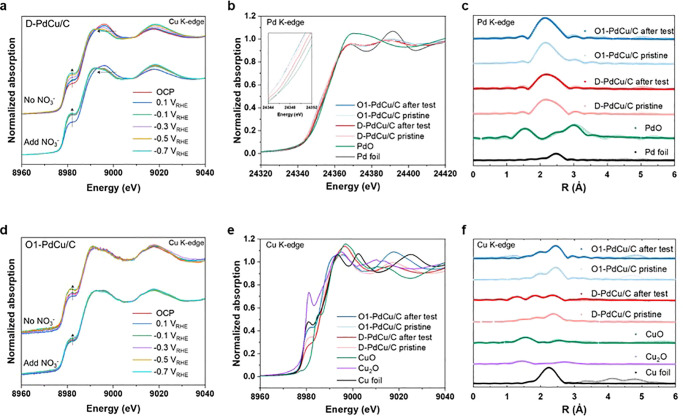
XAS analysis of D-PdCu/C and O1-PdCu/C catalysts. The in situ XAS measurement of Cu K-edge for (a) D-PdCu/C and (d) O1-PdCu/C during NO_3_RR in absence and presence of NO_3_^–^ ions in 0.05 M Na_2_SO_4_ electrolyte. Normalized XANES at the (b) Pd K-edge and (e) Cu K-edge for D-PdCu/C and O1-PdCu/C catalysts before and after long-term electrolysis tests. The corresponding FT-EXAFS spectra of the (c) Pd K-edge and (f) Cu K-edge derived from (b) and (e).

The structurally ordered O1-PdCu/C catalyst which featured both BCC(110) and FCC(111) phase structures exhibited high NO_3_^–^ conversion (98%), high N_2_ selectivity (92%), and was stable throughout the entire test period. After long-term electrolysis, ex situ XAS analysis proved that the ordered structure of PdCu remained the same and the oxidation state of copper was Cu_2_O. The disordered structure exhibited a higher oxidation state of Cu (CuO), resulting in severe dissolution of the copper and loss of catalyst. The intermetallic structure of PdCu provides superior interactions between the Cu and Pd sites, which contributes to excellent stability during the electrochemical conversion of nitrate.

## Methods

### Electrochemical Performance Test on Ordered PdCu Catalysts

Electrochemical NO_3_RR and NO_2_RR were measured using the three-electrode electrochemical cell (Pine Research Instrumentation) connected with a potentiostat. We prepared catalyst inks to deposit on a glassy carbon electrode as a working electrode for measurement by ring disk electrode (RDE). Each catalyst was dissolved into each solvent, which is an addition of Nafion ionomer solution (5 wt %, 20 μL mL^–1^) in a mixture of ultrapure water and isopropanol acolohol (1.5 mg mL^–1^). The Pd metal loading on the GCE was set to 15 μg_*Pd*_ cm^–2^ confirmed by ICP-MS and the deposited ink was dried under ambient conditions. Ag/AgCl (saturated potassium chloride) and Pt wire were selected as a reference electrode and counter electrode, resepectively. All reported potentials were reversible hydrogen electrode (RHE) calibrated by using hydrogen gas purging into the electrolyte and Pt electrode. We collected all LSV curves after electrochemical surface cleaning by sweeping potentials from −1.0 to 0.4 V_*RHE*_ for 200 cycles in a 0.05 M Na_2_SO_4_ solution. To avoid the possible attached hydrogen bubble produced by HER during the NO_3_RR and NO_2_RR measurement, we rotated the RDE at 1000 rpm and operated the LSV experiments at a scan rate of 50 mV s^–1^. The LSV experiment was initiated at 0.2 V_*RHE*_ and repeated in a potential range of −1.0 to 0.4 V_*RHE*_. Ar gas was continuously purged in the electrolyte to establish inert conditions by eliminating any oxygen that might remain in the electrolyte. For screening of NO_3_RR and NO_2_RR activity for all catalysts, we added 20 mM NO_3_ or 2 mM NO_2_ into 0.05 M Na_2_SO_4_ electrolyte and collected the LSV data from the first LSV curve. During the 50 cycle operation, no significant changes of LSV curves were detected. We examined the possible ion and gaseous products for selectivity of all catalysts by using electrolysis tests in a two-chamber electrolytic cell that connected with the potentiostat and in situ mass spectrometry. The working electrode (0.25 cm^2^) and Ag/AgCl reference electrode were placed in the cathode chamber under Ar condition. The Pt wire counter electrode was placed in the anode chamber. The electrolyte in the cathode was 0.05 M Na_2_SO_4_ + 100 ppm of NO_3_^–^–N electrolyte, and the anode chamber was 0.05 M Na_2_SO_4_ electrolyte. We measured the NO_3_^–^ conversion yield, and selectivity of N_2_, NO_2_^–^, and NH_4_^+^ for D-PdCu/C and O1-PdCu/C catalysts in different applied potentials through chronoamperometry (CA) at −0.1, −0.3, −0.5, and −0.7 V_*RHE*_. After the electrolysis for 24 h at each applied potential, we measured remaining NO_3_^–^–N from the initial 100 ppm of NO_3_^–^–N in the electrolyte to calculate the NO_3_^–^ conversion yield. Long-term electrolysis for stability testing was conducted at −0.5 V_*RHE*_ electrolysis for 24 h and repeated for 20 cycles (total of 480 h electrolysis).

### Product Analysis

Titration technique was used to quantify the ion concentrations of NO_3_^–^–N, NO_2_^–^–N, and NH_4_^+^–N. The extracted small amount of solutions from the electrolysis were properly diluted and placed into the thin-layer quarts cuvettes to measure the adsorption intensity detected by the ultraviolet–visible (UV–Vis) spectrophotometer. Each calibration curve was collected from each adsorption intensity and wavelength (345 nm for NO_3_^–^–N, 540 nm for NO_2_^–^–N, and 655 nm for NH_4_^+^–N). The details for preparing color agents and each ion product measurements were described in our previously reported paper.^35,36^ We attached an in situ mass spectrometer (Cirrus 2, MKS Instruments) to the sealed chamber cell for investigating the gaseous products. After stabilizing the gas analyzer signal, we operated the gas analyzer during the electrolysis purged by ultra high purity Ar carrier gas at a 20 mL min^–1^.

### Calculation of NO_3_^–^ Conversion Rate and N_2_ Selectivity


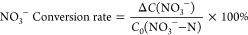
1
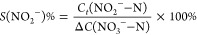
2
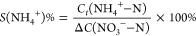
3
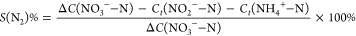
4Δ*C*_0_(NO_3_^–^–N) is the difference of NO_3_^–^ concentrations between before and after the electrolysis tests, and *C*_0_ is the initial NO_3_^–^ concentration. *C*_*t*_ is a change of concentration after the electrochemical reduction time, and N_2_ selectivity is estimated from the total nitrogen mass balance, considering NO_3_^–^–N, NO_2_^–^–N, and NH_4_^+^–N as main products. The electrolyte volume in the cathode compartment is 20 mL of 0.05 M Na_2_SO_4_ including 100 ppm of NO_3_^–^–N.

## Supporting Information Available

Additional experimental details, materials, data, and characterizations, including XRD, morphology, ECSAs, ICP-MS, electrochemical performance, product distributions, in situ mass spectrometer, and XAS analyses (PDF)
