# Supplementary material for: Atomically Ordered PdCu Electrocatalysts for Selective and Stable Electrochemical Nitrate Reduction
Source: ACS Energy Lett. 2023 Oct 19;8(11):4746–52. doi: 10.1021/acsenergylett.3c01672 (PMC10644382; doi:10.1021/acsenergylett.3c01672)
Supplement: Supplementary file 1 — nz3c01672_si_001.pdf [file nz3c01672_si_001.pdf]

Supplementary Information for Atomically ordered PdCu electrocatalysts for selective and stable electrochemical nitrate reduction

Jeonghoon Lim,[†] David A. Cullen,[‡] Eli Stavitski,[¶] Seung Woo Lee,[*,†] and Marta C. Hatzell[*,†,§]

†*Woodruff School of Mechanical Engineering, Georgia Institute of Technology, Atlanta, GA, 30332 USA*

‡*Center for Nanophase Materials Sciences, Oak Ridge National Laboratory, Oak Ridge, Tennessee 37831, United States*

¶*National Synchrotron Light Source II, Brookhaven National Laboratory, Upton, New York 11973, United States*

§ *School of Chemical and Biomolecular Engineering, Georgia Institute of Technology, Atlanta, GA, 30332 USA*

E-mail: seung.lee@me.gatech.edu; marta.hatzell@me.gatech.edu

Materials.

Palladium(II) acetylacetonate ($Pd(acac)_2$, 99%), copper(II) acetylacetonate ($Cu(acac)_2$, ≥99%), L-Ascorbic acid ($C_6H_8O_6$, 99%), and oleylamine (OA, $C_{18}H_{37}N$, 70%) were used for synthesizing PdCu bimetallic nanoparticles. Sodium sulfate (Na_2SO_4, ≥99%), sodium nitrate ($NaNO_3$, ≥99%), and sodium nitrite ($NaNO_2$, ≥99%) were utilized as neutral pH electrolytes, nitrate, and nitrite sources. Phosphoric acid (H_3PO_4, ≥85%), sulfanilamide ($C_6H_8N_2O_2S$, ≥98%), and N-(1-Naphthyl) ethylenediamine dihydrochloride ($C_{12}H_{14}N_2$, ≥98%) were used for a color agent for nitrite measurement. Sodium hydroxide (NaOH, ≥97%), salicylic acid ($C_7H_6O_3$, ≥99%), sodium citrate dihydrate ($C_6H_5Na_3O_7 \cdot 2H_2O$, ≥99%), sodium nitroferricyanide(III) dihydrate ($C_5FeN_6Na_2O \cdot 2H_2O$, ≥99%), and sodium hypochlorite solution (NaClO, ≥98%) were considered as the indophenol blue method for ammonium measurement. Above all materials were purchased from Sigma-Aldrich. Nitarte TNTplus test kit was obtained from HACH and Vulcan XC-72R carbon support was purchased from Cabot Corporation. Nafion membrane was obtained from Fuel Cell Store Company. Ultrapure water (18.2 MΩ) was purified from Millipore Milli-Q.

Synthesis of ordered PdCu bimetals.

The procedure to synthesize the ordered structure of PdCu comprises two steps. First, we prepared randomly disordered PdCu bimetallic nanoparticles supported by Vulcan XC-72R carbon (D-PdCu/C) by means of simple one-pot method. The D-PdCu/C catalyst was synthesized without using any capping agents and surfactants. In detail, 15.2 mg of Pd $(acac)_2$, 26.6 mg of Cu $(acac)_2$, 71.2 mg of L-ascorbic acid (AA), 13 mL of oleylamine (OA), and 40 mg of Vulcan XC-72R were used as Pd and Cu precursors, reducing agent, solvent, and carbon support. After a solvothermal reduction process at 200 °C for 12 h, the disordered PdCu alloy catalyst was obtained and the bimetal NPs were uniformly dispersed on the carbon support. We conducted various heat treatments from 200 to 500 °C for 1 h under the reductive atmosphere (4 % H_2, and 96 % Ar flow) to search the atomically ordered

nanoparticles of Pd and Cu.

Synthesis of Cu nanoparticles on carbon (Cu NP/C)

For the procedure to synthesize the Cu nanoparticles on carbon support, 20 wt% targeted amount of Cu $(acac)_2$, 71.2 mg of L-ascorbic acid (AA), 13 mL of oleylamine (OA), and 40 mg of Vulcan XC-72R were used as Cu precursors, reducing agent, solvent, and carbon support. After a solvothermal reduction process at 200 °C for 12 h, the Cu NPs were uniformly dispersed on the carbon support, and we collected the samples by centrifugation (Supplementary Fig. 13).

Characterization of ordered PdCu catalysts.

X-ray powder diffraction (XRD) patterns were collected using PANalytical Empyrean XRD system for crystalline identification with Cu Kα radiation (λ = 1.54051 $\mathring{A}$). TEM images were obtained by FEI Tecnai F20 at 200 kV operation. The aberration corrected high-angle annular dark-field scanning TEM (HAADF-STEM) combined with complementary energy dispersive X-ray spectroscopy (EDS) was performed on a JEOL NEOARM operated at 200 kV. All TEM grid samples were prepared by drop casting on a gold grid (Electron Microscopy Science Co.), and then dried under ambient conditions. Inductively coupled plasma mass spectrometer (ICP-MS, PerkinElmer Elan DRC) was used to determine the ratio of Pd and Cu atoms, and measure the leaching concentrations of Pd and Cu after long-term electrolysis. The X-ray absorption spectroscopy (XAS) spectra at Pd k-edge and Cu k-edge were measured from 8-ID (ISS) Beamline National Synchrotron Light Source II at Brookhaven National Laboratory, using a Si (111) double crystal monochromator and a passivated implanted planar silicon fluorescence detector at room temperature, with energy calibrated using Pd foil and Cu foil. The catalyst samples were sealed in Kapton films for ex-situ XAS measurements and our customizing designed cell was used for in-situ XAS measurements. All X-ray absorption near edge structure (XANES) and extended X-ray absorption fine struc-

ture (EXAFS) data were analyzed using the Athena and Artemis software for conversion of raw data to µ(E) spectra, background subtraction and normalization, Fourier transformation and plotting, and fitting in k-space and R-space.

ECSA calculation.

The ECSA_{PdO} values were calculated from the integration of the Pd oxide reduction region around 0.6 V_{RHE} in Fig. 2c. This is the most extended approach to determine ECSA of Pd-based catalysts.[1,2] We also estimated the ECSAs for all catalysts measured by TEM measurement. We counted the diameter of nanoparticles (d_i) over 200 individual nanoparticles and measured the number(l_i)-averaged particle sizes, d_N.

$$\mathrm{d}_N = \frac{\sum_i^n l_i \cdot d_i}{\sum_i^n l_i} \tag{S1}$$

We used d_S for the surface averaged diameter and metal density (ρ_{metal}) of Pd (12.02 g cm^{-3}) and Cu (8.96 g cm^{-3}) to calculate ECSA_{TEM}, assuming all nanoparticles have a spherical shape.

$$\text{ECSA}_{TEM} = \frac{6}{\rho_{metal} \cdot d_S} \tag{S2}$$

$$\mathrm{d}_S = \frac{\sum_i^n l_i \cdot d_i^3}{\sum_i^n l_i \cdot d_i^2} \tag{S3}$$

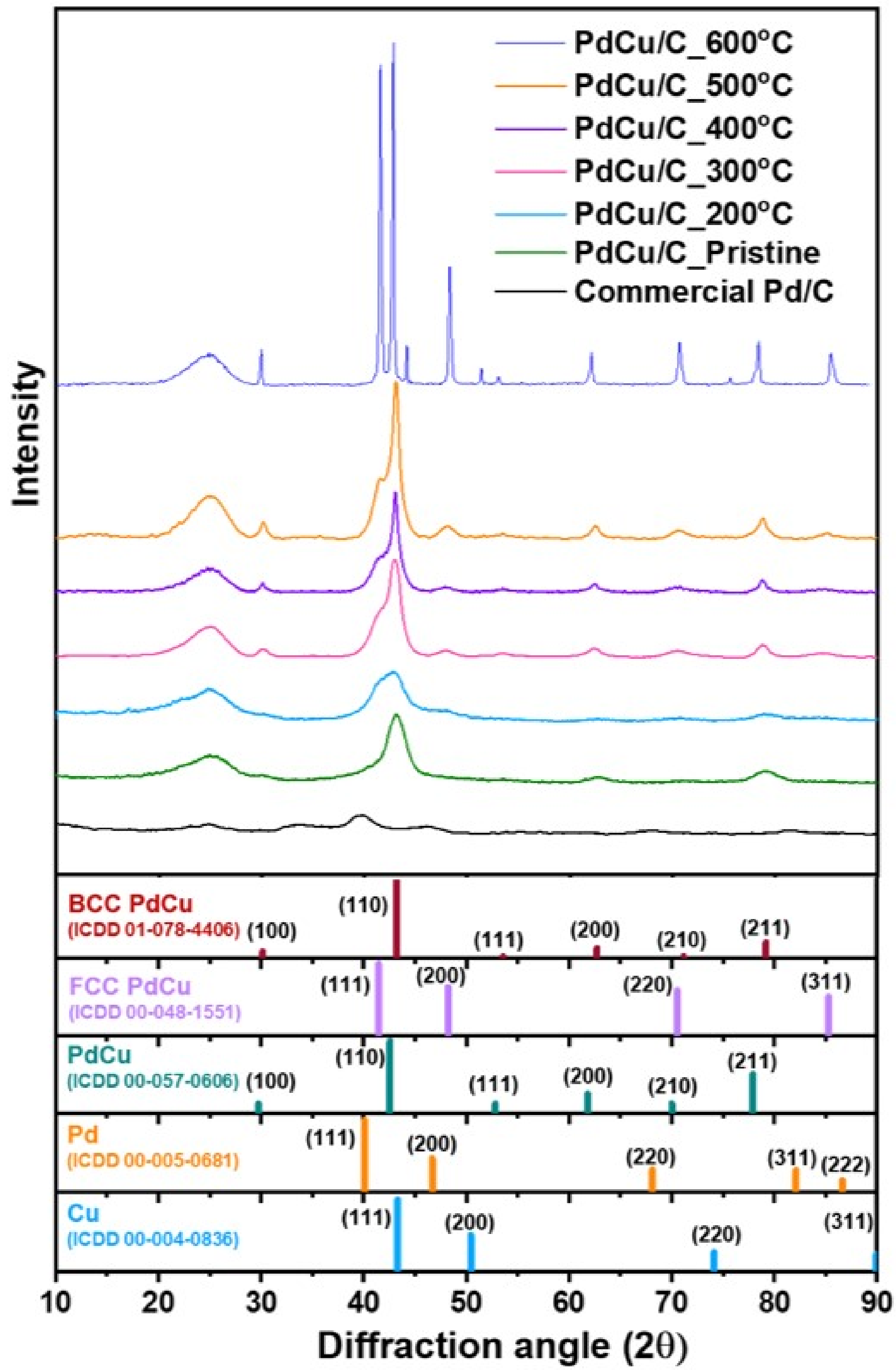

Supplementary Figure 1: XRD patterns of commercial Pd/C and PdCu/C annealed at 200 °C, 300 °C, 400 °C, 500 °C, and 600 °C. The higher temperature annealing conditions exhibited the larger NP size and more FCC(111) phase.

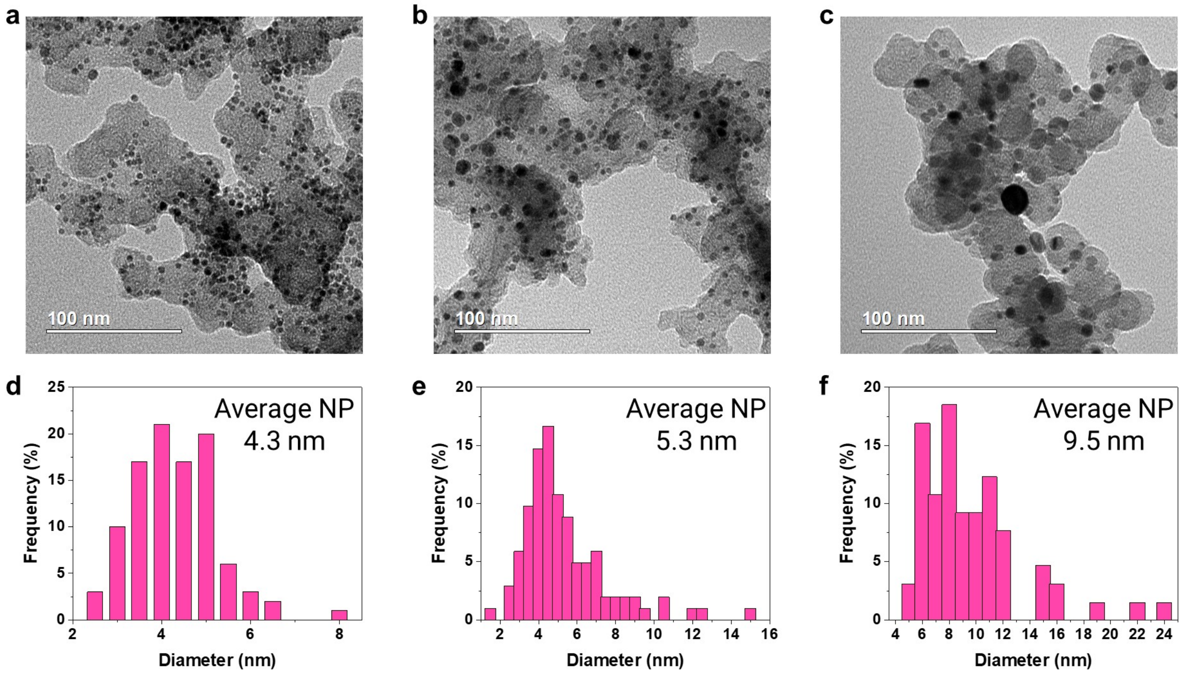

Supplementary Figure 2: Morphology and NP size distribution of (a, d) D-PdCu/C pristine, (b, e) O1-PdCu/C (annealed at 500 °C), and (c, f) O2-PdCu/C (annealed at 600 °C). The number of NPs for measuring average size of NP is over 200 NPs.

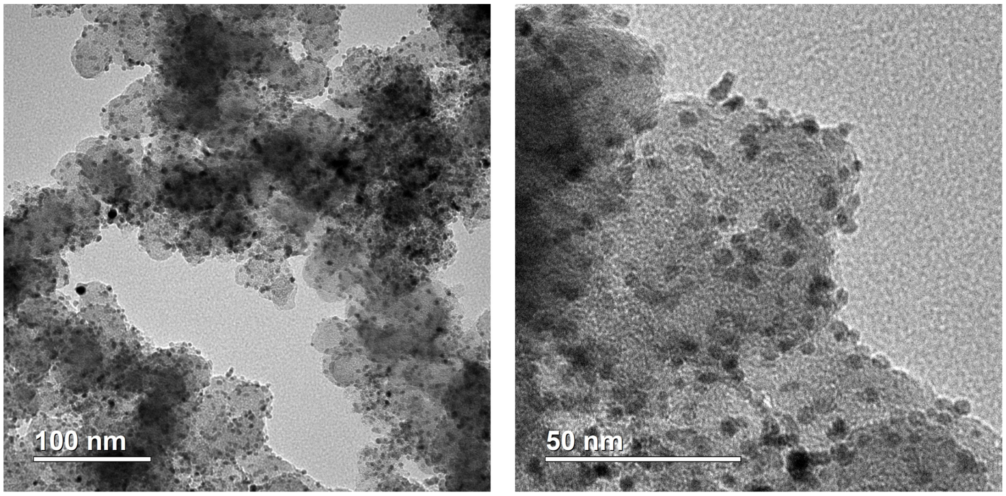

Supplementary Figure 3: TEM images of the commercial Pd/C (Fuel Cell Store Co.). The average NP size is 3-5 nm.

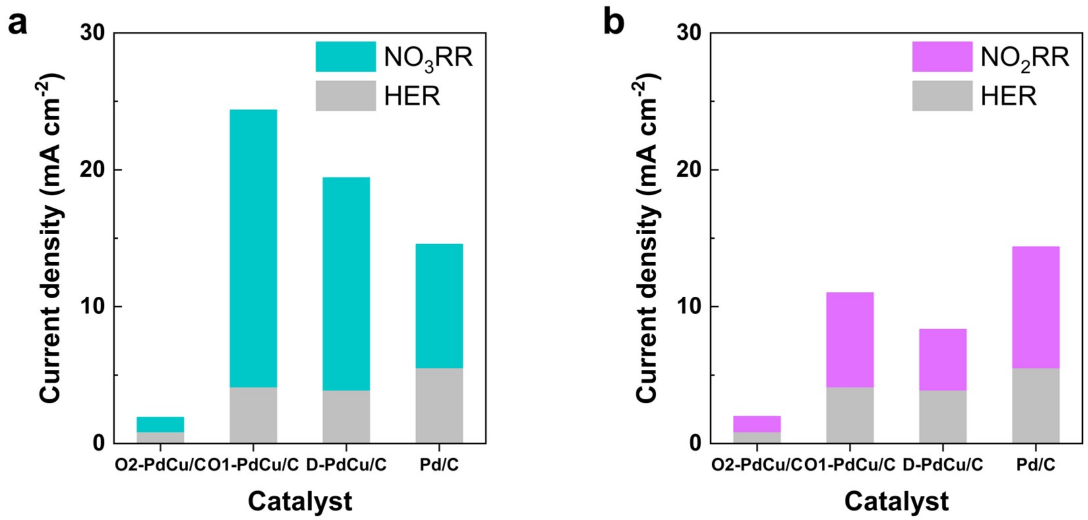

Supplementary Figure 4: Summary of NO_3RR, NO_2RR and HER activity for O2-PdCu/C, O1-PdCu/C, D-PdCu/C and commercial Pd/C catalysts. We collected a partial current density at -0.5 V_{RHE}.

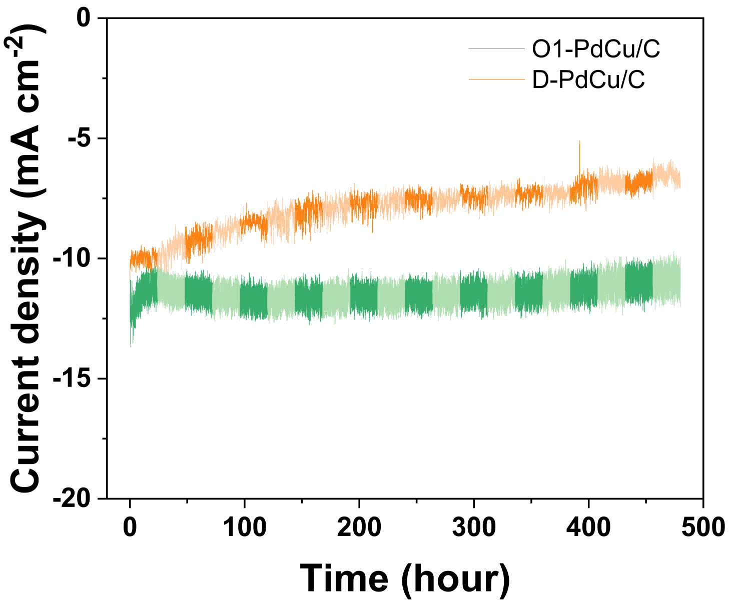

Supplementary Figure 5: Current density of O1-PdCu/C and D-PdCu/C electrodes over time using the chronoamperometry (CA). Each measurement is 24 h electrolysis at -0.5 V_{RHE} applied potential in an Ar-purged 0.05 M Na_2SO_4 + 100 ppm NO_3^--N electrolyte up to 20 consecutive cycles (480 hours).

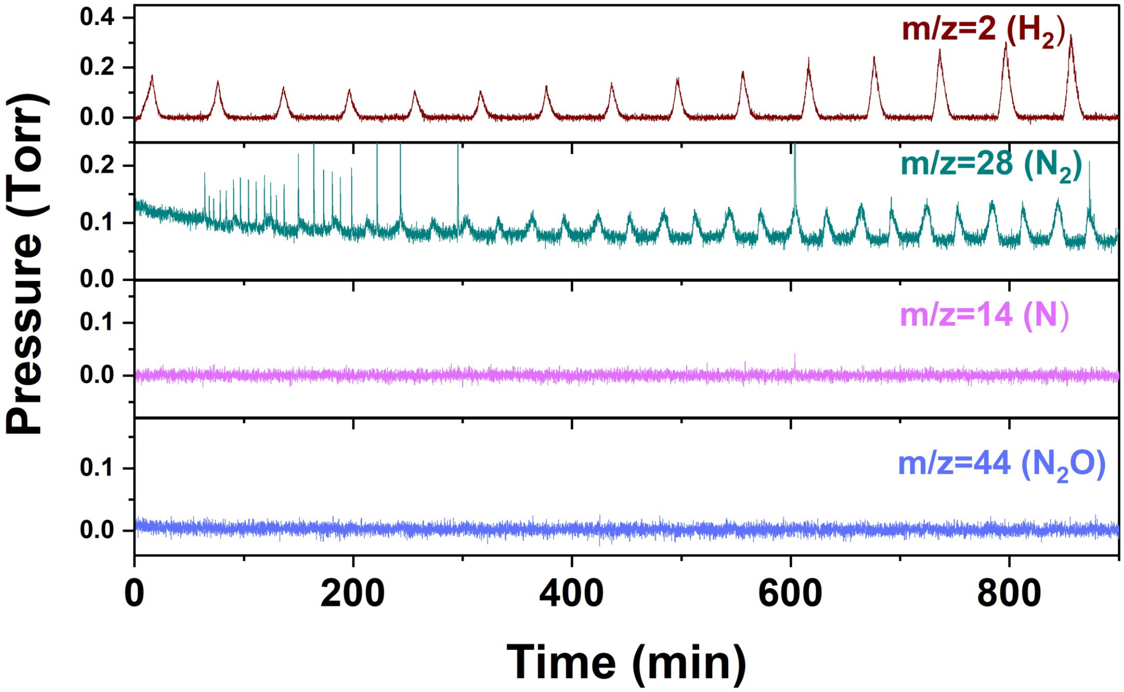

Supplementary Figure 6: In-situ mass spectrometer result of O1-Cu Pd NC/C showed that H_2 and N_2 were detected as gaseous products (CA at -0.5 V_{RHE} in 0.05 M Na_2SO_4 + 100 ppm of nitrate-N electrolyte).

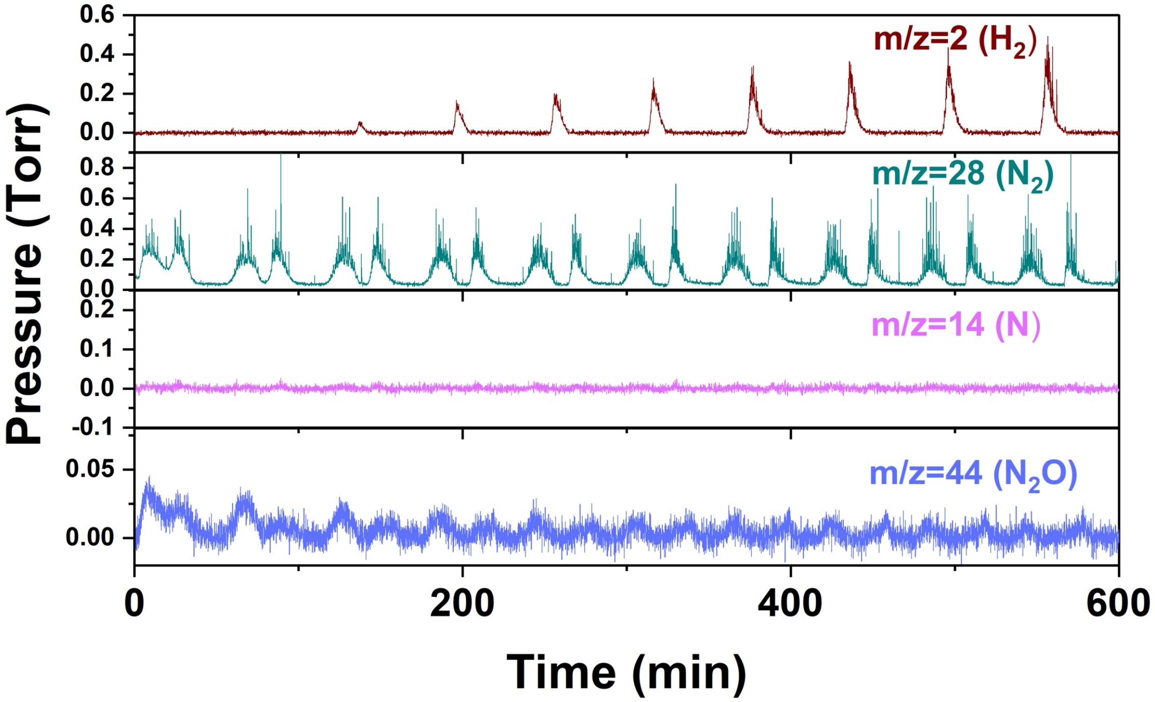

Supplementary Figure 7: In-situ mass spectrometer result of D-PdCu/C showed that H_2, N_2, and N_2O were detected as gaseous products (CA at -0.5 V_{RHE} in 0.05 M Na_2SO_4 + 100 ppm of nitrate-N electrolyte).

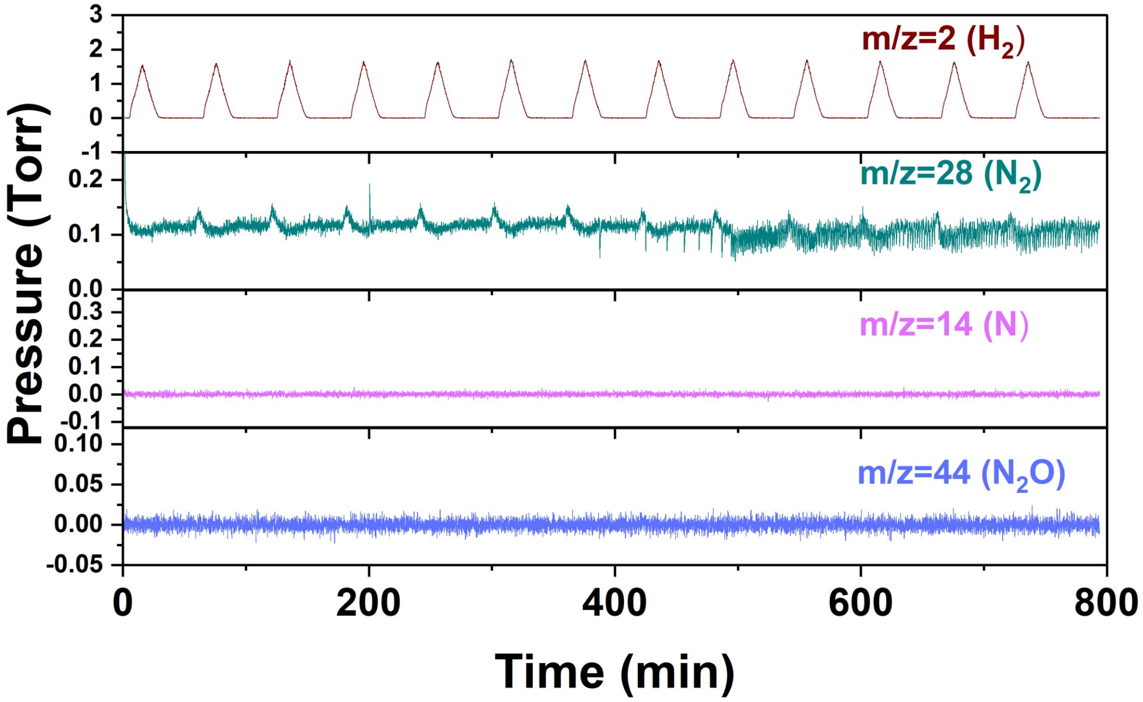

Supplementary Figure 8: In-situ mass spectrometer result of commercial Pd/C showed that H_2, and N_2 were detected as gaseous products (CA at -0.5 V_{RHE} in 0.05 M Na_2SO_4 + 100 ppm of nitrate-N electrolyte).

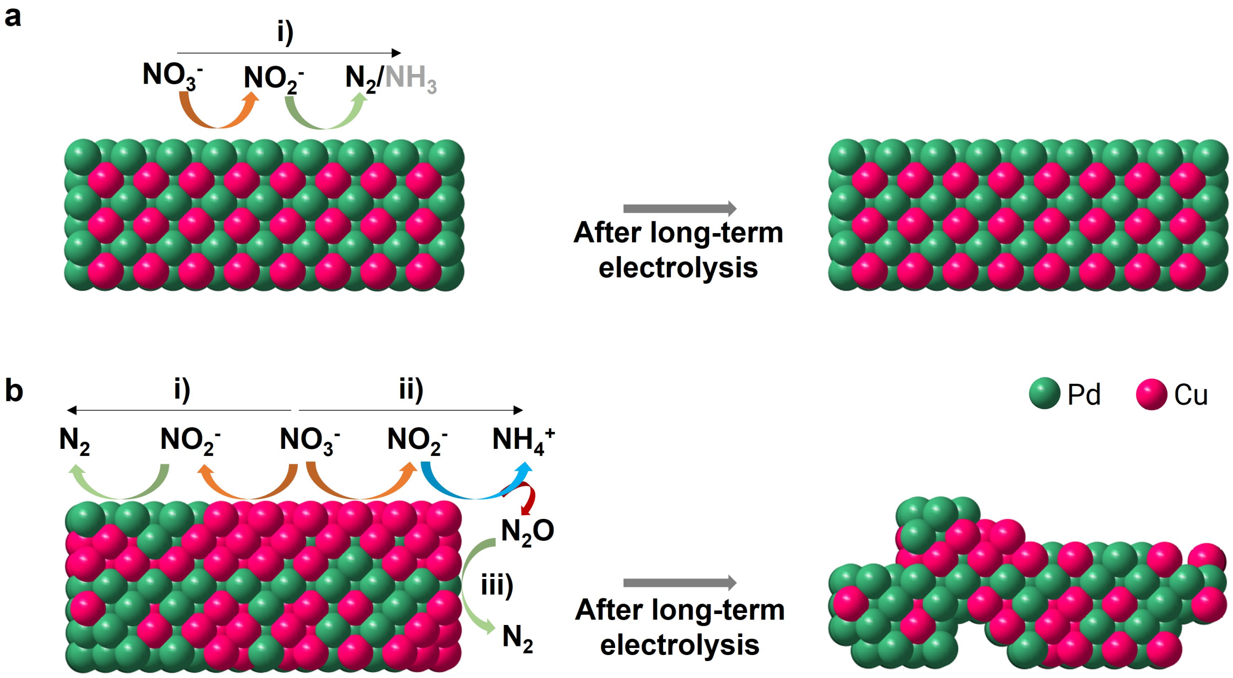

Supplementary Figure 9: A proposed pathway for the reduction of NO_3^- to N_2 and NH_4^+; (a) O1-PdCu/C, and (b) D-PdCu/C.

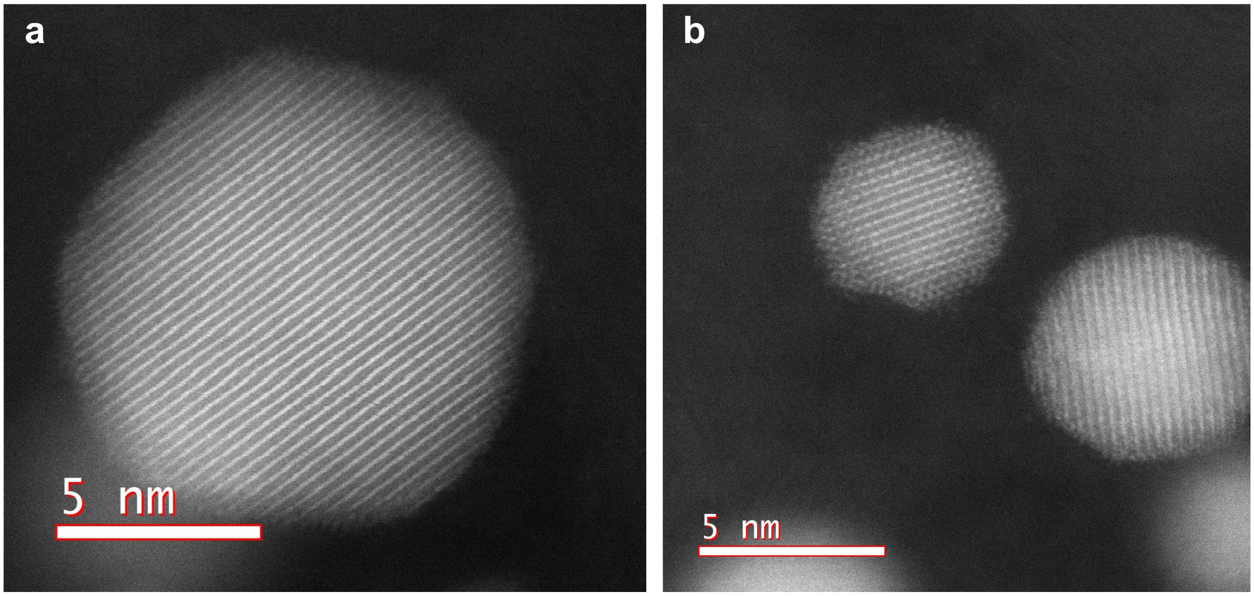

Supplementary Figure 10: STEM images of O1-PdCu/C after repeated 20 cycles (480 electrolysis).

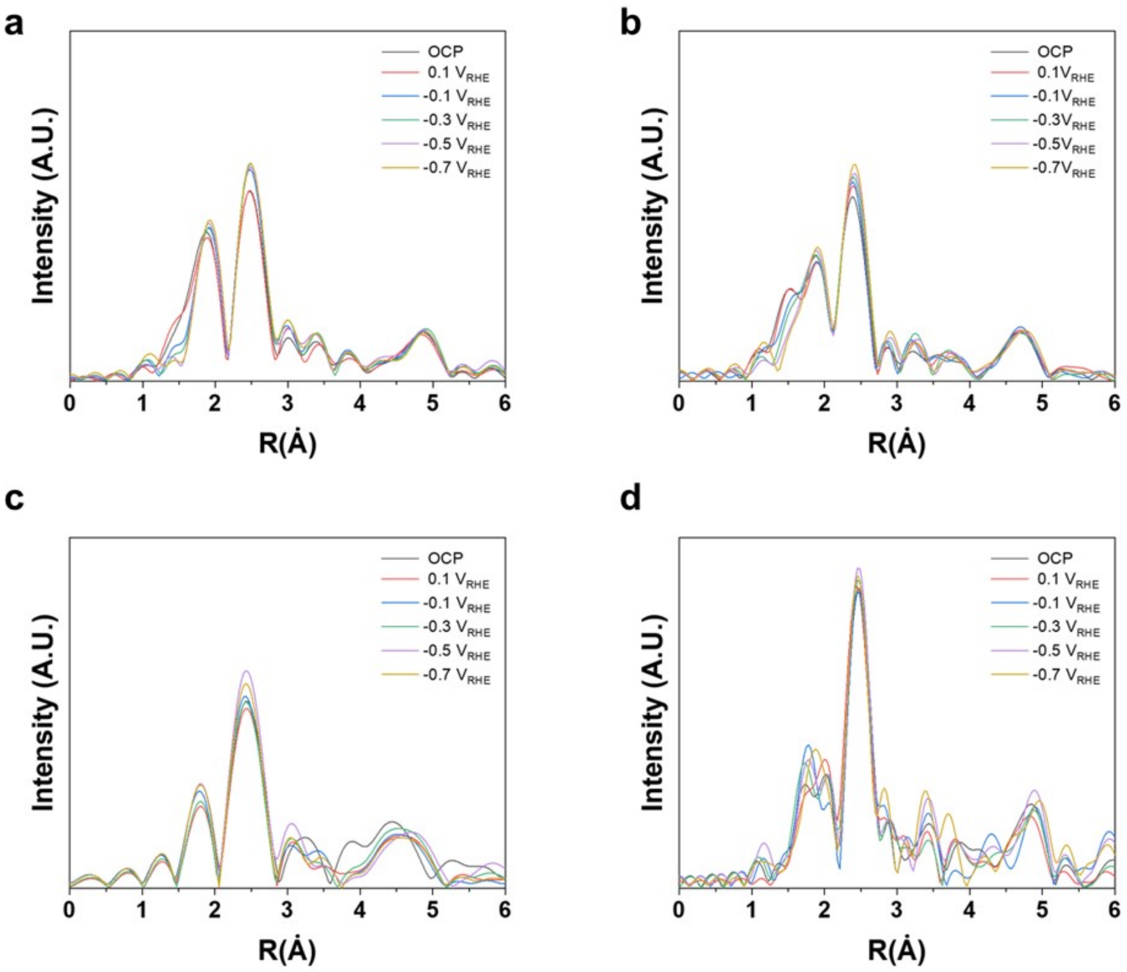

Supplementary Figure 11: The in-situ Fourier transformed Cu K-edge EXAFS spectra. (a) D-PdCu/C in electrolyte including NO_3^-, (b) D-PdCu/C in electrolyte excluding NO_3^- , (c) O1-PdCu/C in electrolyte including NO_3^-, and (d) O1-PdCu/C in electrolyte excluding NO_3^-.

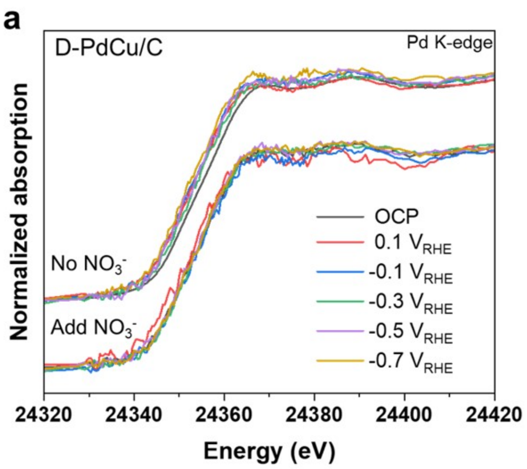

Supplementary Figure 12: The in-situ Fourier transformed Pd K-edge EXAFS spectra for (a) D-PdCu/C and (b) O1-PdCu/C.

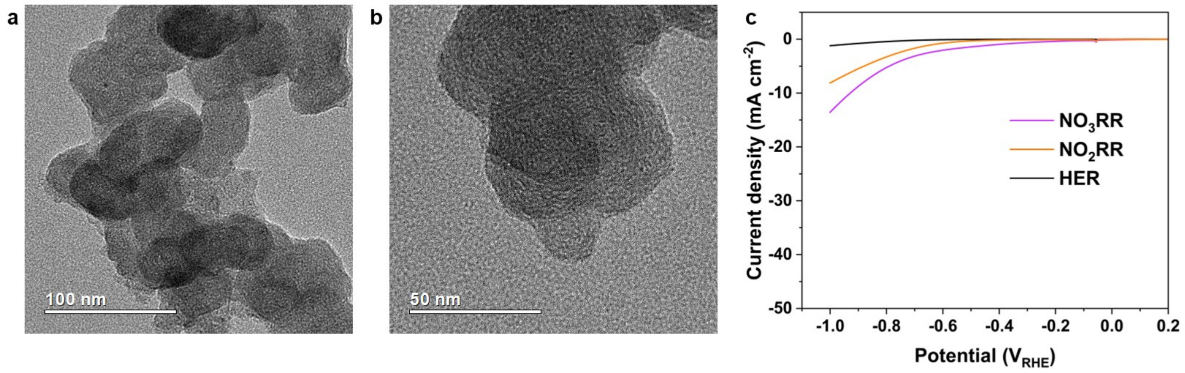

Supplementary Figure 13: TEM images of Cu nanoparticles on Vulcan carbon XC72R support (Cu NP/C) (a, b), and NO_3RR activity, NO_2RR activity, and HER of Cu NP/C (c). The average of Cu nanoparticle size is 2.04 nm.

Supplementary Table 1: ICP-MS results for pristine powder of O2-PdCu/C, O1-PdCu/C and D-PdCu/C catalysts.

Catalyst	Pd at%	Cu at%
O2-PdCu/C	52.3	47.7
O1-PdCu/C	49.9	50.1
D-PdCu/C	48.1	51.9

Supplementary Table 2: ECSA of commercial Pd/C, D-PdCu/C, O1-PdCu/C, and O2-PdCu/C catalysts.

	Commercial PD/C	D-PdCu/C	O1-PdCu/C	O2-PdCu/C
Average NP size [nm]	3.2	4.3	5.3	9.5
$ECSA_{PdO}$ [m^2 g^{-1}_{Catal}]	55.6	33.0	28.5	12.8
$ECSA_{PdO}$ [m^2 g^{-1}_{Pd}]	76.3	45.4	39.2	17.5
$ECSA_{TEM}$ [m^2 g^{-1}_{Catal}]	149	114	72.1	41.1

Supplementary Table 3: Leaching concentration of Pd and Cu in electrolytes after 20 repeated stability tests of O1-PdCu/C and D-PdCu/C electrodes measured by ICP-MS (unit: ppb).

Electrode	Pd	Cu
O1-PdCu/C	4.64	42.3
D-PdCu/C	18.2	547

Supplementary Table 4: Recently reported Pd-Cu electrocatalysts for denitrifcation.

Catalyst	Electrolyte	Analysis	Potential (V_{RHE})	Time	Yield	Ref.
**O1-PdCu/C**	**0.05 M Na_2SO_4**	**mass balance**	**CA -0.5**	**24 h**	**NO_3 conv. 98%**	**This**
	**100 ppm nitrate-N**	**in-situ mass spectrometry**		**20 cycles**	**N_2 92%**	**work**
$Cu_{28}Pd_{72}$ Nanowires	0.05 M Na_2SO_4	mass balance	CA -0.2	4 h	NO_3 conv. 98%	3
	22.5 ppm nitrate-N				N_2 76%	
Cu/Pd on Ni foam	0.05 M Na_2SO_4	mass balance	CA -0.6	24 h	NO_3 conv. 92%	4
	100 ppm nitrate-N				N_2 84%	
Cu-Pd@N-OMC-600	0.1 M Na_2SO_4	mass balance	CA -0.6	24 h	NO_3 conv. 97%	5
	100 ppm nitrate-N				N_2 99%	
Pd-Cu/PNC	0.1 M Na_2SO_4	mass balance	CA -0.6	24 h	NO_3 conv. 97%	6
	100 ppm nitrate-N				N_2 83%	
PdCu NCs-NOMC	0.1 M Na_2SO_4	mass balance	CA -0.7	24 h	NO_3 conv. 86%	7
	0.1 M $NaNO_3$			10 cycles	N_2 60%	
Pd_4Cu_4@N-pC	N_2 containing neutral	mass balance	CA -0.6	24 h	NO_3 conv. 95%	8
	simulated sewage				N_2 80%	
CuPd@rGO	0.1 M Na_2SO_4	mass balance	CA -0.6	12 h	NO_3 conv. 97%	9
	100 ppm nitrate-N				N_2 86%	
PdCu@OMC	0.1 M Na_2SO_4	mass balance	CA -0.6	24 h	NO_3 conv. 29%	10
	500 ppm nitrate-N				N_2 74%	

Supplementary Table 5: The best Pd EXAFS fitting parameters for Pd foil, PdO, D-PdCu/C pristine, D-PdCu/C after test, O1-PdCu/C pristine, and O1-PdCu/C after test.

**Sample**	**Path**	**CN**	**R(Å)**	σ^2(**Å**^{2}) **X 10**$^{-3}$	Δ **E**$_0$ **(eV)**	**R-factor (%)**
Pd foil	Pd-Pd1	**12**	2.74±0.01	6.6±0.3	-1.1±0.9	1.7
	Pd-Pd2	**6**	3.80±0.03	10.0±3.6		
PdO	Pd-O	**4**	2.02±0.01	0.3±0.8	6.5±1.4	0.3
	Pd-Pd1	**4**	3.07±0.01	5.5±0.5		
	Pd-Pd2	**8**	3.44±0.01	5.5±0.5		
D-PdCu/C pristine	Pd-Cu	2.4	2.55±0.01	3.7±0.5	-0.4±2.6	0.7
	Pd-Pd	3.0	2.75±0.03	13.0±2.6		
D-PdCu/C after test	Pd-Cu	2.2	2.52±0.03	1.1±2.4	-3.3±6.1	0.5
	Pd-Pd	2.8	2.72±0.05	5.6±3.6		
O1-PdCu/C pristine	Pd-Cu	3.4	2.54±0.01	3.6±0.6	-3.0±1.8	1.5
	Pd-Pd	2.7	2.70±0.02	12.1±3.6		
O1-PdCu/C after test	Pd-Cu	3.4	2.55±0.01	3.4±0.7	-2.0±2.2	2.0
	Pd-Pd	2.8	2.72±0.03	13.4±4.7		

* The value of S_0^2 determined for the Pd foil was estimated as 0.8. The EXAFS fitting range is listed below: Pd foil; R range (1.2 – 3.5 Å) and k-range (3.0 – 12.0 Å), PdO; R range (1.2 – 3.5 Å) and k-range (3.0 – 12.0 Å), D-PdCu/C; R range (1.25 – 3.0 Å) and k-range (3.0 – 12.0 Å^{-1}), O1-PdCu/C; R range (1.25 – 3.0 Å) and k-range (3.0 – 12.0 Å^{-1}). **Bold numbers** indicate fixed coordination number of each path according to the theoretical crystal structure based on FEFF calculations.

Supplementary Table 6: The best Cu EXAFS fitting parameters for Cu foil, Cu_2O, CuO, D-PdCu/C pristine, D-PdCu/C after test, O1-PdCu/C pristine, and O1-PdCu/C after test.

**Sample**	**Path**	**CN**	**R(Å)**	σ^2**(Å^2) X 10^{-3}**	**Δ E$_0$ (eV)**	**R-factor (%)**
Cu foil	Cu-Cu1	**12**	2.54±0.01	7.6±0.4	-3.1±1.3	1.9
	Cu-Cu2	**6**	3.60±0.01	7.6±0.4		
Cu_2O	Cu-O1	1.1±0.4	1.83±0.01	2.1±2.7	0.15±2.5	1.7
	Cu-Cu	**2**	2.99±0.02	11.1±2.0		
	Cu-O_2	3.2±2.2	3.49±0.04	5.0±7.3		
CuO	Cu-O1	**2**	1.95±0.02	2.0	6.0±1.9	0.2
	Cu-O2	**0.5**	2.06±0.07	9.1±23.3		
	Cu-Cu1	**4**	2.91±0.02	9.0±1.3		
	Cu-Cu2	**4**	3.11±0.02	9.6±2.0		
D-PdCu/C pristine	Cu-O	1.8	1.91±0.06	10.7±6.1	-7.2±4.4	1.7
	Cu-Cu	6	2.51±0.05	29.6±8.6		
	Cu-Pd	2.4	2.55±0.02	4.0±0.7		
D-PdCu/C after test	Cu-O	3	1.86±0.17	14.7±15.6	-9.3±20.0	0.9
	Cu-Cu1	4.2	2.49±0.24	11.4±22.9		
	Cu-Pd	2.2	2.50±0.23	4.8±9.9		
	Cu-Cu2	4.2	2.76±0.31	12.1±24.1		
O1-PdCu/C pristine	Cu-O	1.2	1.77±0.19	23.1±30.4	-8.0±3.0	1.2
	Cu-Cu	4.8	2.59±0.07	29.1±12.8		
	Cu-Pd	3.4	2.56±0.01	4.5±0.5		
O1-PdCu/C after test	Cu-O	1.2	1.88±0.12	16.4±17.0	-6.7±3.2	1.3
	Cu-Cu	4.7	2.59±0.13	36.9±25.7		
	Cu-Pd	3.4	2.57±0.01	4.8±0.5		

* The value of S_0^2 determined for the Cu foil was estimated as 0.8. The EXAFS fitting range is listed below: Cu foil; R range (1.25 – 3.3 Å) and k-range (3.0 – 12.0 Å), Cu_2O; R range (1.2 – 3.3 Å) and k-range (2.7 – 11.0 Å), CuO; R range (1.25 – 3.0 Å) and k-range (3.0 – 11.0 Å^{-1}), D-PdCu/C; R range (1.2 – 3.0 Å) and k-range (3.0 – 11.0 Å^{-1}), O1-PdCu/C; R range (1.25 – 3.0 Å) and k-range (3.0 – 11.0 Å^{-1}). **Bold numbers** indicate fixed coordination number of each path according to the theoretical crystal structure based on FEFF calculations.

References

(1) Khan, I. A.; Khan, L.; Khan, S. I.; Badshah, A. Shape-control synthesis of PdCu nanoparticles with excellent catalytic activities for direct alcohol fuel cells application. *Electrochimica Acta* **2020**, *349*, 136381.

(2) Wang, T.-J.; Li, F.-M.; Huang, H.; Yin, S.-W.; Chen, P.; Jin, P.-J.; Chen, Y. Porous Pd-PdO Nanotubes for Methanol Electrooxidation. *Advanced Functional Materials* **2020**, *30*, 2000534.

(3) Fu, C.; Shu, S.; Hu, L.; Liu, Z.; Yin, Z.; Lv, X.; Zhang, S.; Jiang, G. Electrocatalytic nitrate reduction on bimetallic Palladium-Copper Nanowires: Key surface structure for selective dinitrogen formation. *Chemical Engineering Journal* **2022**, *435*, 134969.

(4) Shen, Z.; Liu, D.; Peng, G.; Ma, Y.; Li, J.; Shi, J.; Peng, J.; Ding, L. Electrocatalytic reduction of nitrate in water using Cu/Pd modified Ni foam cathode: High nitrate removal efficiency and N2-selectivity. *Separation and Purification Technology* **2020**, *241*, 116743.

(5) Wang, J.; Teng, W.; Ling, L.; Fan, J.; Zhang, W.-x.; Deng, Z. Nanodenitrification with bimetallic nanoparticles confined in N-doped mesoporous carbon. *Environmental Science: Nano* **2020**, *7*, 1496–1506.

(6) Gu, T.; Teng, W.; Bai, N.; Chen, Z.; Fan, J.; Zhang, W.-x.; Zhao, D. Nano-spatially confined Pd–Cu bimetals in porous N-doped carbon as an electrocatalyst for selective denitrification. *Journal of Materials Chemistry A* **2020**, *8*, 9545–9553.

(7) Xu, H.; Xu, H.; Chen, Z.; Ran, X.; Fan, J.; Luo, W.; Bian, Z.; Zhang, W.-x.; Yang, J. Bimetallic PdCu nanocrystals immobilized by nitrogen-containing ordered mesoporous carbon for electrocatalytic denitrification. *ACS applied materials & interfaces* **2019**, *11*, 3861–3868.

(8) Chen, M.; Wang, H.; Zhao, Y.; Luo, W.; Li, L.; Bian, Z.; Wang, L.; Jiang, W.; Yang, J. Achieving high-performance nitrate electrocatalysis with PdCu nanoparticles confined in nitrogen-doped carbon coralline. *Nanoscale* **2018**, *10*, 19023–19030.

(9) Chen, Z.; Wang, Y.; Wang, J.; Hu, Z.; Teng, W.; Fan, J.; Zhang, W.-x. Enhanced activity and selectivity of electrocatalytic denitrification by highly dispersed CuPd bimetals on reduced graphene oxide. *Chemical Engineering Journal* **2021**, *416*, 129074.

(10) Fan, J.; Xu, H.; Lv, M.; Wang, J.; Teng, W.; Ran, X.; Gou, X.; Wang, X.; Sun, Y.; Yang, J. Mesoporous carbon confined palladium–copper alloy composites for high performance nitrogen selective nitrate reduction electrocatalysis. *New Journal of Chemistry* **2017**, *41*, 2349–2357.
